# A *GAMYB* homologue *CsGAMYB1* regulates sex expression of cucumber via an ethylene-independent pathway

**DOI:** 10.1093/jxb/eru176

**Published:** 2014-04-30

**Authors:** Yan Zhang, Xiaolan Zhang, Bin Liu, Wenjiao Wang, Xingwang Liu, Chunhua Chen, Xiaofeng Liu, Sen Yang, Huazhong Ren

**Affiliations:** ^1^College of Agronomy and Biotechnology, China Agricultural University, Beijing 100193, P. R. China; ^2^Beijing Key Laboratory of Growth and Developmental Regulation for Protected Vegetable Crops, China Agricultural University, Beijing 100193, P. R. China

**Keywords:** *CsGAMYB1*, cucumber, ethylene, *GAMYB*, gibberellin, sex expression.

## Abstract

Cucumber (*Cucumis sativus* L.) is a typical monoecious vegetable with individual male and female flowers, and has been used as a model plant for sex determination. It is well known that sex differentiation of cucumber can be regulated by phytohormones, such as gibberellic acid (GA) and ethylene. The molecular mechanism of female sex expression modulated by ethylene has been widely understood, but how GA controls male sex expression remains elusive. In hermaphroditic *Arabidopsis* and rice, GA can regulate stamen and anther development via the transcriptional regulation of *GAMYB*. Here we characterized a *GAMYB* homologue *CsGAMYB1* in cucumber. We found that *CsGAMYB1* is predominantly expressed in male flower buds, where its expression is upregulated by GA_3_ treatment. CsGAMYB1 protein is localized in the nucleus. *CsGAMYB1* can partially rescue stamen development and fertility phenotypes of an *Arabidopsis myb33 myb65* double mutant. However, constitutive overexpression of *CsGAMYB1* in wild-type *Arabidopsis* resulted in male sterility, which mimics the effect of GA overdose in flower development. Knockdown of *CsGAMYB1* in cucumber decreases the ratio of nodes with male and female flowers, and ethylene is not involved in this process. Our data suggest that *CsGAMYB1* regulates sex expression of cucumber via an ethylene-independent pathway.

## Summary

We find that *CsGAMYB1*, a positive regulator of GA signalling, can regulate sex expression of cucumber. This provides a new insight into the mechanism of GA in sex determination.

## Introduction

Gibberellins (GAs), one kind of endogenous growth regulator, play an essential role in reproductive development of plants, especially in staminate development (Aya *et al.*, 2009; Bai and Xu, 2013; King and Evans, 2003; Pharis and King, 1985; Plackett *et al.*, 2011; Song *et al.*, 2013). For example, GA application can promote development of male flowers in cucumber (*Cucumis sativus* L.) (Pike and Peterson, 1969; Wittwer and Bukovac, 1962). Additionally, GA-deficient mutants of *Arabidopsis* and tomato (*Solanum lycopersicum*) display abnormal stamen, and anther and pollen development leading to male sterility (Cheng *et al.*, 2004; Goto and Pharis, 1999; Jacobsen and Olszewski, 1991; Koornneef and van der Veen, 1980; Nester and Zeevaart, 1988).

Recently, several studies have demonstrated that GA regulates staminate development via the GA signalling pathway (Aya *et al.*, 2009; Cheng *et al.*, 2004; Fleet and Sun, 2005; Sun, 2010; Sun, 2011). In this pathway, GA first binds the GID1 receptor and promotes binding of DELLA proteins (repressors of GA action, and plant growth and development) to GID1; this GA–GID1–DELLA complex then enables the rapid degradation of DELLA proteins by the proteasome, which releases the inhibitory effect of the DELLA proteins and allows GA action to occur (Fleet and Sun, 2005; Harberd *et al.*, 2009; Murase *et al.*, 2008; Plackett *et al.*, 2014; Sun, 2010; Sun, 2011; Ueguchi-Tanaka *et al.*, 2007). GAMYB, a positive regulator involved in the GA signalling pathway, has been known to act as an important downstream component in the degradation of DELLA proteins (Achard *et al.*, 2004; Fleet and Sun, 2005; Olszewski *et al.*, 2002). *GAMYB* was first identified in barley (*Hordeum vulgare*) aleurone cells, where its expression is upregulated by GA treatment (Gubler *et al.*, 1995). HvGAMYB can bind specifically to GA-response elements in promoter regions of an α-amylase gene and other GA-regulated genes encoding hydrolytic enzymes, and constitutive expression of *HvGAMYB* mimics the positive effects of exogenous GA application on the transcriptional activation of these genes (Cercos *et al.*, 1999; Gubler *et al.*, 1999). *GAMYB* has also been demonstrated to play an important role in flower development, especially in anther development. For example, *HvGAMYB* shows high expression levels in barley anthers, where it is also upregulated by GA_3_. Overexpression of *HvGAMYB* in barley results in decreased anther length and male sterility (Murray *et al.*, 2003), which phenocopies those plants treated with excessive exogenous GA (Colombo and Favret, 1996). In rice, loss-of-function mutations of *GAMYB* lead to abnormal anther and pollen development (Kaneko *et al.*, 2004; Liu *et al.*, 2010). In addition, *OsGAMYB* is also involved in GA-mediated programmed cell death (PCD) of tapetal cells, exine, and Ubisch body formation, and microarray analysis revealed that *OsGAMYB* can modulate most GA-regulated gene expression in rice anthers (Aya *et al.*, 2009).

In *Arabidopsis*, there is a small family of *GAMYB*-like genes (Stracke *et al.*, 2001), in which *AtMYB33*, *AtMYB65*, and *AtMYB101* were identified to be able to substitute for barley and rice *GAMYB* in transactivating the α-amylase promoter (Gocal *et al.*, 2001). Expression pattern analysis found that *AtMYB33*, *AtMYB65*, and *AtMYB101* have a predominant expression in floral shoot apices and flowers, and the expression of *AtMYB33* can be induced by exogenous GA (Achard *et al.*, 2004; Gocal *et al.*, 2001; Millar and Gubler, 2005). To further understand the function of *AtGAMYBs*, the deficient mutants for *AtMYB33* and *AtMYB65* were isolated, and the double mutant *myb33 myb65* displays the phenotypes of shorter filaments, pollen abortion, and male sterility (Millar and Gubler, 2005). Furthermore, neither *myb33* nor *myb65* showed an abnormal phenotype compared with wild-type plants, suggesting that *AtMYB33* and *AtMYB65* are functionally redundant (Millar and Gubler, 2005). Taken together, these observations suggest that *GAMYB* is involved in GA-regulated stamen and anther development.

Cucumber is a typical monoecious vegetable with unisexual flowers, and has been served as a model plant for sex determination and differentiation (Malepszy and Niemirowicz-Szczytt, 1991). In young floral buds of cucumber, both stamen primordia and carpel primordia are initiated, and sex determination occurs just after the bisexual stage; subsequently, male or female flowers are formed and enlarged owing to the selective arrestment of carpel or stamen development, respectively (Bai *et al*., 2004; Malepszy and Niemirowicz-Szczytt, 1991). In this process, ethylene treatment can produce increased numbers of female flowers in cucumber (Iwahori *et al.*, 1969; MacMurray and Miller, 1968), and the mechanism has been widely understood. Two major genes encoding ACC synthase (a key enzyme of ethylene biosynthesis), *F* (*CsACS1G*), and *M* (*CsACS2*) control female sex expression in cucumber; the *F* gene governs the development of female flowers (Knopf and Trebitsh, 2006; Mibus and Tatlioglu, 2004; Trebitsh *et al.*, 1997), whereas the *M* gene inhibits stamen development in flower buds (Bie *et al.*, 2013; Li *et al.*, 2012; Saito *et al.*, 2007; Wang *et al.*, 2010; Yamasaki *et al.*, 2001; Yamazaki *et al.*, 2003). In addition, GA application can promote the male tendency (Pike and Peterson, 1969), but the molecular regulation remains elusive. Previous studies have confirmed that GA can modulate stamen and anther development via the transcriptional regulation of *GAMYB* in hermaphroditic plants, such as *Arabidopsis* and rice. However, in monoecious species cucumber whether *GAMYB* is involved in GA-regulated male tendency in the process of sex differentiation or not is still unknown. Therefore, in this study, a *GAMYB* orthologous gene in cucumber, designated as *CsGAMYB1*, was identified, and its spatial and temporal expression patterns were characterized. *CsGAMYB1* is predominantly expressed in male flower buds, where its expression is upregulated by exogenous GA_3_ application, and CsGAMYB1 protein is localized in the nucleus. Ectopic expression of *CsGAMYB1* can partially rescue the phenotypes of *myb33 myb65* double mutant in *Arabidopsis*; however, constitutive overexpression of *CsGAMYB1* in wild type resulted in male sterility. Furthermore, we generated *CsGAMYB1*-RNAi transgenic plants in cucumber and found that reduced transcript levels of *CsGAMYB1* can result in decreased ratio of nodes with male and female flowers, but no effect on ethylene production and expression of *F* and *M* genes. Our results indicate that *CsGAMYB1* can regulate ethylene-independent sex expression of cucumber.

## Materials and methods

### Plant materials and growth conditions

Monoecious cucumber (*Cucumis sativus L*.) line 3407 was used in this study. The seeds were germinated on wet filter paper in a Petri dish at 28 °C in dark overnight. Then the resulting seedlings were grown in a growth chamber under 16h/8h with 25 °C/18 °C in day/night. Upon the two true-leaf stage, plants were transferred to a greenhouse. The A*rabidopsis* mutant *myb33 myb65* (Columbia background) was provided by Millar’s lab (Millar and Gubler, 2005), and Columbia (Col) was used as a wild-type control. *Arabidopsis* seeds were germinated on Murashige-Skoog (MS) medium, which contains 1% sucrose and 0.2% phytagar, at 4 °C for 3 d and then moved to 22 °C under a regime of 16h light/8h dark. Seedlings were transferred to soil 7–10 d after germination. For GA_3_ treatment, male flower buds of cucumber were sprayed with 200 μm GA_3_ (and mock-sprayed with 0.1% ethanol). Expression analyses were done after 4h of treatment.

### Cloning of *CsGAMYB1*

Total RNA was extracted from cucumber leaves using Promega’s SV Total RNA Isolation System, and cDNA was synthesized using MultiScribe^TM^ reverse transcriptase (Applied Biosystems). The cDNA samples were amplified by PCR: 95 °C for 5min, 30 cycles of 95 °C for 30s, 52 °C for 30s, and 72 °C for 2.5min, and then 72 °C for 10min. The primers are listed in supplementary material Table S3 available at *JXB* online.

### Sequence alignment and phylogenetic analysis

Through BLAST analysis in Phytozome (http://www.phytozome.net/search.php) or the *Arabidopsis* Information Resource (http://www.Arabidopsis.org) using the sequence information of CsGAMYB1 protein, the amino acid sequence of related GAMYB proteins in various species were obtained. The multiple sequence alignment of CsGAMYB1 and related GAMYB proteins was performed using ClustalW in the MEGA5 software package, and the boxes were drawn using the BoxShade web site (http://www.ch.embnet.org/software/BOX_form.html). The phylogenetic tree was constructed using the Neighbor–Joining (NJ) method (Saitou and Nei, 1987) with Poisson model and 1000 bootstrap replicates test through MEGA5 software.

### Expression analysis by qRT-PCR

Total RNA was extracted using Promega’s SV Total RNA Isolation System, and cDNA was synthesized using MultiScribe^TM^ reverse transcriptase (Applied Biosystems). Quantitative real-time RT-PCR (qRT-PCR) was performed using SYBR^®^ *Premix Ex Taq* ^TM^ from TaKaRa (China) on an Applied Biosystems 7500 real-time PCR system. The cucumber *α-TUBULIN* (*TUA)* and *Arabidopsis actin2* were used as internal controls in analysing gene expression in cucumber and *Arabidopsis*, respectively. And three biological replicates were performed for these experiments. The gene specific primers for qRT-PCR are listed in Supplementary Table S3 available at *JXB* online.

### *In situ* hybridization

Shoot apex of 10-day-old seedlings, and male and female flower buds from 45-day-old cucumbers grown in the greenhouse were fixed, embedded, sectioned, and hybridized as described (Zhang *et al.*, 2013). Digoxigenin-labelled sense and antisense RNA probes were generated using SP6 and T7 RNA polymerase (Roche) through PCR amplification respectively. The primer pairs are listed in Supplementary Table S3 available at *JXB* online.

### Subcellular localization in onion epidermal cells

For transient expression in onion epidermal cells, the full-length coding region of *CsGAMYB1* was cloned and fused to the upstream of the green fluorescent protein (GFP) between the *Eco*RI and *Bam*HI sites in the pEZS-NL vector (http://deepgreen.stanford.edu) to generate *35S:GFP–CsGAMYB1*; the empty pEZS-NL vector was used a control. The onion epidermal layers were prepared and bombarded, as previously described (Varagona *et al.*, 1992), with gold particles containing the plasmid using a Bio-Rad PDS-1000/He particle delivery system. After bombardment, the onion epidermises were placed on MS medium and incubated in darkness at 22 °C for 24h. Fluorescence signals were detected using Olympus BX 51 fluorescence microscopy. The primer sequences used for vector construction are listed in Supplementary Table S3 available at *JXB* online.

### Transformation of *Arabidopsis*

To make the *CsGAMYB1* overexpression construct, full-length *CsGAMYB1* cDNA was cloned and inserted into the pCAMBIA1305.1 vector between the *Bgl*II and *Spe*I sites. The construct was then introduced into *Agrobacterium* by electroporation and transformed into Col or *myb33 myb65* plants as described (Clough and Bent, 1998). The transgenic plants were screened on MS medium with 25mg l^–1^ hygromycin. The primers for overexpression construct are listed in Supplementary Table S3 available at *JXB* online.

### Transformation of cucumber

To generate the CsGAMYB1-RNAi transgenic plants of cucumber, two fragments of CsGAMYB1 were amplified using specific primers containing *Asc*I (5’ end) and *Swa*I (3’ end) sites, and *Spe*I (5’ end) and *Bam*HI (3’ end) sites. The two fragments were inversely inserted into the pFGC1008 vector, and the resulting *CsGAMYB1*-RNAi construct was then introduced into *Agrobacterium* by electroporation and transformed into the monoecious cucumber line 3407 using the cotyledon transformation method as described previously (Wang *et al.*, 2014). The primers for RNAi construct are listed in Supplementary Table S3 available at *JXB* online.

### Quantification of ethylene

To examine the ethylene production from cucumber, shoot apices were excised at the 4-leaf stage. The samples were enclosed in a 10ml vessel after weighing, and sealed with a rubber stopper. After incubation at 25 °C for 0.5h, 1ml of gas was extracted using a syringe and injected into a gas chromatograph (GC-9A, Shimadzu, Japan). Ethylene was quantified using an activated alumina column and hydrogen flame ionization detector (FID). Standard ethylene gas was used for calibrating the instrument.

## Results

### Identification of the *CsGAMYB1* gene from cucumber

**Fig. 1.**
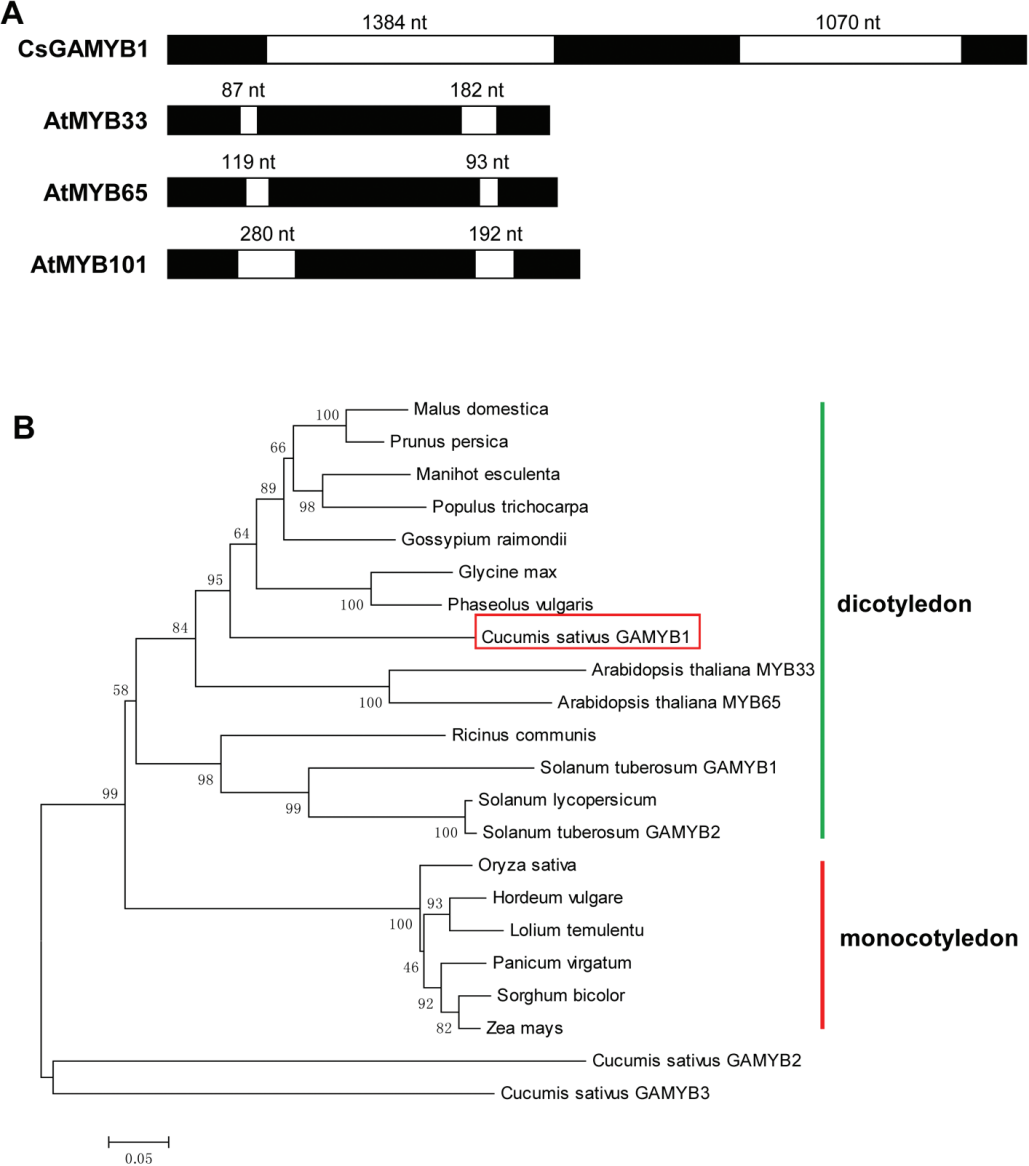
Structural and phylogenetic analyses of GAMYB homologues in various species. (A) Structural analysis of *GAMYB* genes in cucumber and *Arabidopsis*. Exons and introns are shown in black and white, respectively. Cs, *Cucumis sativus*; At, *Arabidopsis thaliana*. (B) Phylogenetic analysis of GAMYB homologues in various species. This phylogenetic tree was constructed using the Neighbor–Joining (NJ) method through MEGA5 software. Eighteen species were used for this analysis and formed two main groups: dicotyledon group and monocotyledon group. A GAMYB homologue from cucumber is indicated in the box. Gene ID for each of the GAMYB protein used for this analysis is listed in the “accession numbers”. (This figure is available in colour at *JXB* online.)

Through BLAST analysis in the Cucumber Genome Database (Huang *et al.*, 2009), we discovered three *GAMYB*-like genes named as *CsGAMYB1* (Csa009014), *CsGAMYB2* (Csa019830), and *CsGAMYB3* (Csa013555). The *CsGAMYB1* gene shows the highest similarity compared with other *GAMYB* orthologues, so *CsGAMYB1* was chosen and analysed in this study. *CsGAMYB1* was cloned using cDNA derived from cucumber leaves. Consistent with three *GAMYB* orthologues in *Arabidopsis* (Achard *et al.*, 2004; Gocal *et al.*, 2001; Millar and Gubler, 2005), *CsGAMYB1* also contains three exons and two introns (Fig. 1A), encoding 552 amino acids. The sequence alignment of the amino acid residues of CsGAMYB1 compared with other members of the GAMYB family was performed using ClustalW in the MEGA5 software package (Tamura *et al.*, 2011). CsGAMYB1, HvGAMYB, AtMYB33, and AtMYB65 share an R2R3 repeat DNA-binding domain in their N-terminal regions (Kranz *et al.*, 1998; Romero *et al.*, 1998; Stracke *et al.*, 2001); over this sequence, CsGAMYB1 shows high identity to HvGAMYB, AtMYB33, and AtMYB65, with 88.46%, 87.5%, and 87.5% identity, respectively. In addition, these proteins also contain three conserved motifs Box 1, Box 2, and Box 3, which are typical structures in the GAMYB family (Supplementary Fig. S1 available at *JXB* online) (Gocal *et al.*, 2001).

To further understand the evolutionary relationship between CsGAMYB1 and other GAMYB homologues, phylogenetic analysis was performed using the Neighbor–Joining (NJ) method (Saitou and Nei, 1987) (Fig. 1B). CsGAMYB1 (shown in the box in Fig. 1B) is placed in the same clade as other GAMYB proteins, whereas CsGAMYB2 and CsGAMYB3 are distinct from this clade, suggesting that CsGAMYB1, but not CsGAMYB2 and CsGAMYB3, belongs to the GAMYB family in cucumber. A phylogenetic tree of the GAMYB family can be divided into two main groups: dicotyledon and monocotyledon. Within the dicotyledon group, GAMYB proteins in cucumber, which belongs to the cucurbitaceae family; *Arabidopsis* of the cruciferae family; kidney bean (*Phaseolus vulgaris*) and soybean (*Glycine max*) of the leguminosae family; and several woody plants such as apple (*Malus domestica*), cassava (*Manihot esculenta*), cotton (*Gossypium raimondii*), *Prunus persica*, and *Populus trichocarpa* all fall into the same clade, indicating that these plants may share a common origin. However, CsGAMYB1 is placed in a distinct group with GAMYB homologues in hermaphroditic species such as *Arabidopsis*, tomato, potato (*Solanum tuberosum*), kidney bean, and soybean, and it is also different from GAMYB groups in other monoecious plants such as cassava, *Populus trichocarpa*, and castor bean (*Ricinus communis*). These observations demonstrated that CsGAMYB1 is a GAMYB homologue in cucumber.

### Expression pattern of *CsGAMYB1* in cucumber

**Fig. 2.**
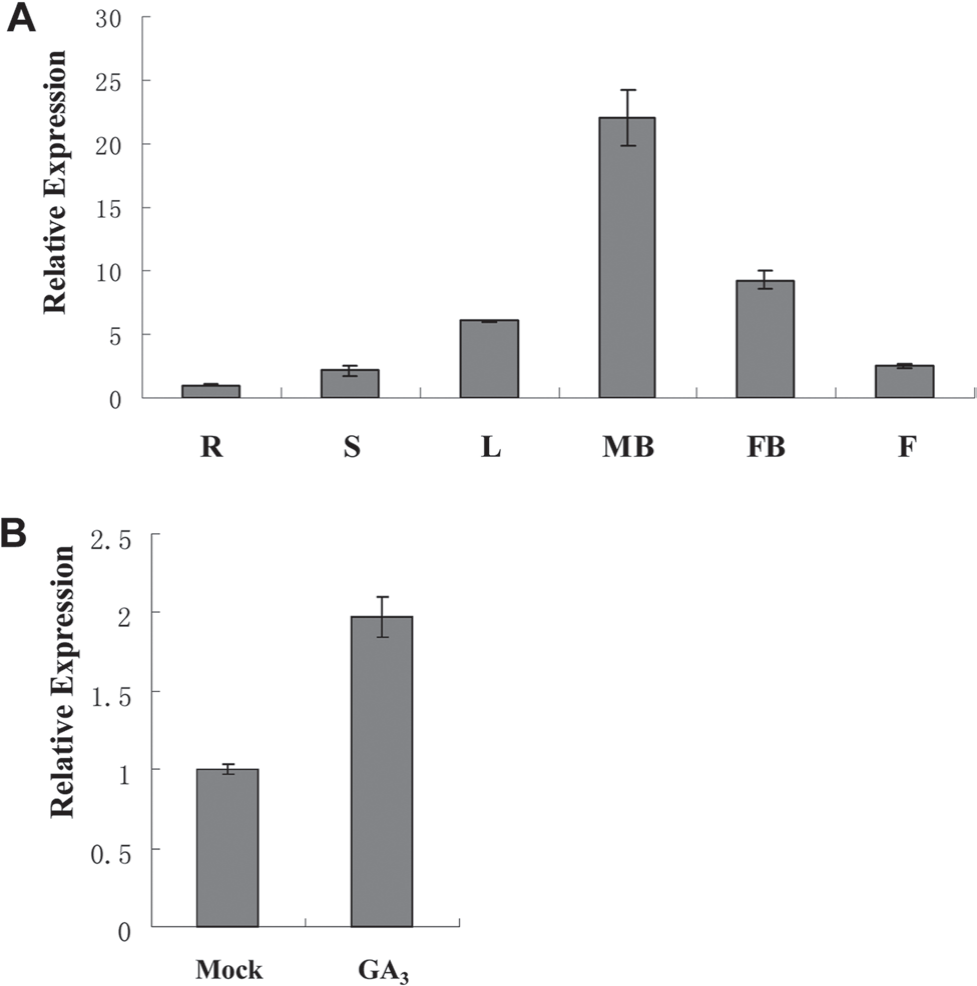
qRT-PCR analyses of *CsGAMYB1* in cucumber. (A) Tissue distribution of *CsGAMYB1* in cucumber. R, root; S, stem; L, leaves; MB, male flower buds; FB, female flower buds; F, fruit. (B) Effect of GA_3_ on the expression of *CsGAMYB1* in male flower buds. Male flower buds were mock-treated with 0.1% ethanol, or treated with 200 μM GA_3_ for 4h. The cucumber *α-TUBULIN* (*TUA*) was used as an internal control, and three biological replicates were performed for these experiments. Error bars indicate the standard errors. (This figure is available in colour at *JXB* online.)

To obtain insights into the biological function of *CsGAMYB1*, we investigated its spatial and temporal expression patterns in cucumber. Quantitative real-time RT-PCR (qRT-PCR) was performed in various cucumber tissues including roots, stems, leaves, male flower buds, female flower buds, and fruits. *CsGAMYB1* was expressed in all examined tissues, and the highest expression was detected in male flower buds (Fig. 2A), where its expression was increased almost two-fold by GA_3_ application (Fig. 2B). These data suggested that *CsGAMYB1* may play an important role in cucumber male flower development.

**Fig. 3.**
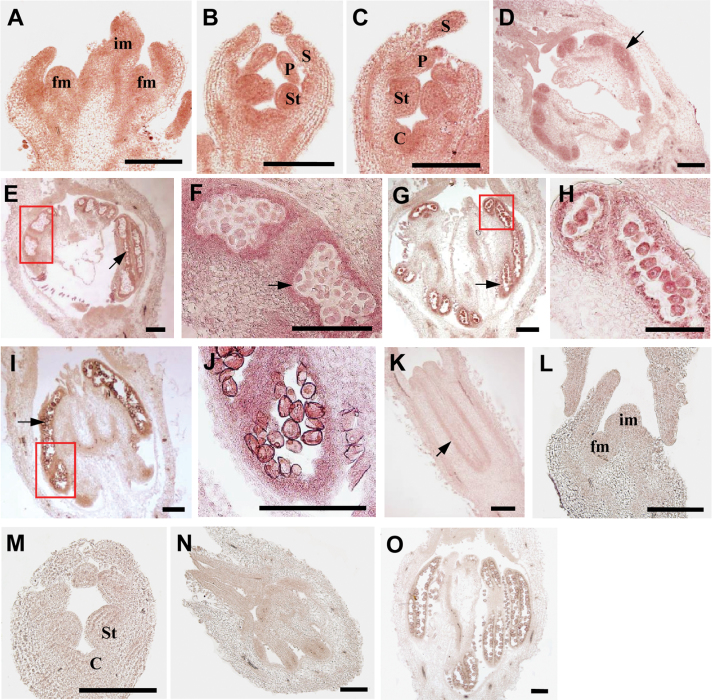
*In situ* hybridization of *CsGAMYB1* during flower development in cucumber. Longitudinal sections of shoot apex (A and L, stage 1), male flower buds at stage 4 (B), stage 5 (C and M), stage 9 (D and N), stage 10 (E), stage 11 (G) and stage 12 (I and O), and female flower buds at stage 8 (K). The morphologies of tetrad in meiosis and pollens in the framed regions of E, G, and I are shown in F, H, and J, respectively. *CsGAMYB1* sense probe hybridizations showed no signals in L–O. The arrows indicate the expression of *CsGAMYB1* in microsporocytes, anther wall, pollen grains or ovary. im, inflorescence meristem; fm, floral meristem; S, sepal; P, petal; St, stamen; C, carpel. Bar=200 μm. (This figure is available in colour at *JXB* online.)

Further, we examined the detailed expression patterns of *CsGAMYB1* during cucumber flower development by *in situ* hybridization (Fig. 3). *CsGAMYB1* RNA was found throughout the inflorescence meristem (im) and floral meristem (fm) in stage 1 of cucumber flower development (Bai *et al*., 2004) (Fig. 3A). When flowers developed into the bisexual stages 4–5, the crucial periods that stamen primordia and carpel primordia initiate, higher expressions were detected in sepal primordia, petal primordia, stamen primordia, and carpel primordia (Fig. 3B, C). For male flowers, during the stages of microsporocytes (stage 9), meiosis (stage 10), uninuclear pollens (stage 11), and mature pollens (stage 12), *CsGAMYB1* was predominately expressed in the microsporocytes (Fig. 3D), anther wall, and pollen grains (Fig. 3E–J). For female flowers, the expression of *CsGAMYB1* was detected in the developing ovary of stage 8 (Fig. 3K), but the signal was weak. As negative controls, *CsGAMYB1* sense probe hybridization showed no signals in the male flowers of stage 1, stage 5, stage 9, and stage 12 (Fig. 3L–O).

### Subcellular localization of CsGAMYB1 protein

**Fig. 4.**
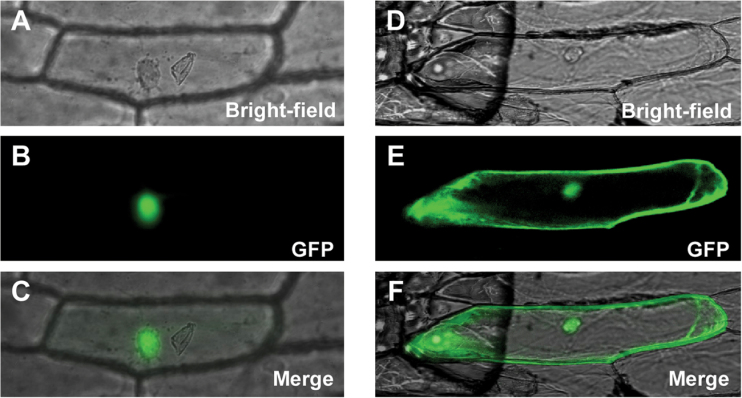
Subcellular localization of CsGAMYB1 protein in onion epidermal cells. 35S:GFP–CsGAMYB1 localized to the nucleus (A–C), whereas 35S:GFP localized throughout the cell (D–F). (This figure is available in colour at JXB online.)

To further determine the subcellular localization of CsGAMYB1 protein, the GFP–CsGAMYB1 fusion protein was constructed under the control of the cauliflower mosaic virus (CaMV) 35S promoter and introduced into onion epidermal cells by particle bombardment. As shown in Fig. 4, the GFP–CsGAMYB1 fusion protein is localized in the nucleus (Fig. 4A–C). As a control, the signals of 35S:GFP are detected throughout the cell (Fig. 4D–F).

### *CsGAMYB1* can partially rescue *myb33 myb65* double mutant phenotypes in *Arabidopsis*

**Fig. 5.**
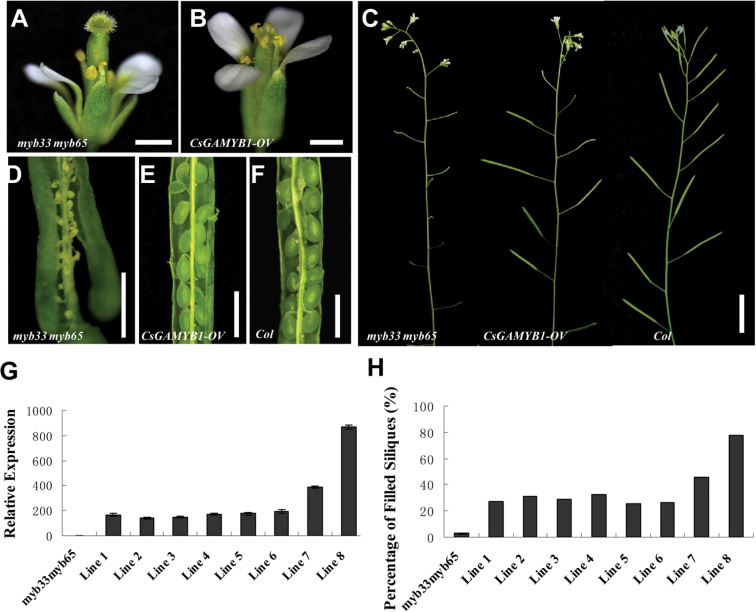
Partial rescue of *myb33 myb65* mutant by ectopic expression of *CsGAMYB1* in *Arabidopsis*. (A, B) Flowers of *myb33 myb65* (A) or *CsGAMYB1* overexpression (B). (C) Inflorescences of *myb33 myb65* (left), *CsGAMYB1* overexpression (middle) or *Col* (right). (D–F) Opened siliques of *myb33 myb65* (D), *CsGAMYB1* overexpression (E) or *Col* (F). Bar=1mm, except C, in which the bar=1cm. (G) qRT-PCR analyses of *CsGAMYB1* in eight selected transgenic lines in the *myb33 myb65* background. The *Arabidopsis actin2* was used as an internal control, and the experiments were repeated in three independent samples. Error bars indicate the standard errors. (H) Fertility in *myb33 myb65* and 8 selected *CsGAMYB1* transgenic lines. The fertility is defined as the percentage of siliques that set seeds per plant. Error bars indicate the standard errors. (This figure is available in colour at *JXB* online.)

To investigate the biological role of *CsGAMYB1*, we ectopically expressed the full-length *CsGAMYB1* cDNA under the control of a 35S promoter in an *Arabidopsis myb33 myb65* double mutant, which displayed shorter filaments, pollen abortion, and male sterility (Millar and Gubler, 2005). A total of 23 independent transgenic lines were obtained, all of which could partially rescue the phenotypes of the *myb33 myb65* double mutant, and all of which displayed similar phenotypes. As shown in Fig. 5, flowers in the transgenic plants had increased filaments length and pollen numbers as compared with those in the *myb33 myb65* double mutant (Fig 5A, B). Consequently, fertility increased in the *CsGAMYB1* transgenic plants (Fig 5C–F). For example, the *myb33 myb65* plants displayed much smaller siliques (Fig 5C), which failed to set any seeds (Fig 5D), whereas ectopic expression of *CsGAMYB1* resulted in normal siliques (Fig 5C), which set seeds with similar shape and numbers, compared with those in wild-type plants (Fig 5E, F). We chose eight transgenic lines to analyse expression of *CsGAMYB1* and plant fertility. The fertility is defined as the percentage of siliques that set seeds per plant. The expression of *CsGAMYB1* displayed different levels in these lines (Fig 5G), although the lines with the higher *CsGAMYB1* mRNA levels showed the higher increase in fertility compared with the *myb33 myb65* plants (Fig 5H). For example, the average fertility of *myb33 myb65* plants was only 2.8%, whereas in the six lines (lines 1, 2, 3, 4, 5, and 6) which had lower *CsGAMYB1* mRNA levels, the average fertility increased to 28.6%. However, in line 7, which had higher *CsGAMYB1* mRNA expression, the fertility reached 46.0%, and the highest *CsGAMYB1* mRNA level in line 8 resulted in 77.3% fertility, which was close to that in wild-type (92.2% fertility; Supplementary Tables S1 and S2 available at *JXB* online), suggesting that *CsGAMYB1* can partially rescue the fertility of the *myb33 myb65* double mutant, but the recovery relies on the levels of *CsGAMYB1* expression. These results implied that the function of *CsGAMYB1* in cucumber, which has unisexual flowers, is conserved with *AtMYB33* and *AtMYB65* in *Arabidopsis*, which has complete flowers with respect to stamen development and plant fertility.

### Constitutive overexpression of *CsGAMYB1* results in male sterility in *Arabidopsis*

**Fig. 6.**
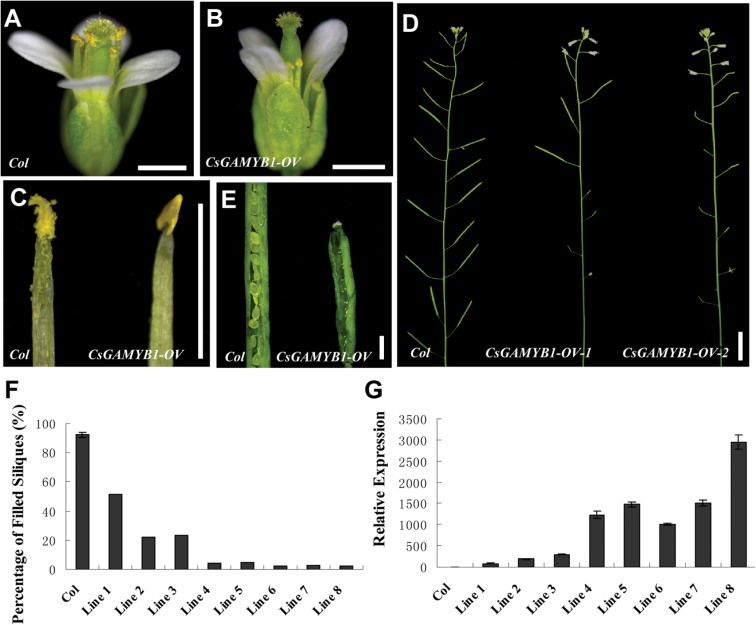
Male sterility in *CsGAMYB1* transgenic plants with high levels of *CsGAMYB1* in *Arabidopsis*. (A and B) Flowers of *Col* (A) or *CsGAMYB1* overexpression (B). (C) Stamens in *Col* (left) or *CsGAIP* overexpression (right). (D) Inflorescences of *Col* (left) or lines of *CsGAMYB1* overexpression (middle and right). (E) Opened siliques of *Col* (left) or *CsGAMYB1* overexpression (right). Bar=1mm, except D, in which the bar=1cm. (F) Fertility in *Col* and 8 selected *CsGAMYB1* transgenic lines. The fertility is defined as the percentage of siliques that set seeds per plant. Error bars indicate the standard errors. (G) qRT-PCR analyses of *CsGAMYB1* in eight selected transgenic lines in the *Col* background. The *Arabidopsis actin2* was used as an internal control, and these experiments were repeated in three independent samples. Error bars indicate the standard errors. (This figure is available in colour at *JXB* online.)

To further explore the function of *CsGAMYB1*, we also generated transgenic lines overexpressing *CsGAMYB1* in *Arabidopsis* wild-type Columbia (Col); a total of 24 independent transgenic lines were obtained. In the transgenic plants, stamens were shorter than those in wild type and failed to fully extend to the pistil (Fig. 6A, B), and anthers were smaller than their wild-type counterparts and failed to generate pollen (Fig. 6C), leading to much smaller siliques, which failed to set any seeds (Fig. 6D, E).

Among the 24 transgenic lines, 8 lines were chosen for further analysis. Of these lines, five lines (lines 4, 5, 6, 7 and 8) were sterile and three other lines (lines 1, 2 and 3) exhibited partial fertility. For example, in these five sterile lines, few siliques that set seeds were present, from which few seeds were obtained, and the average fertility was only 3.1%. However, there were some normal siliques in lines 1, 2, and 3, characterized by the similar seeds number per silique compared with that of wild-type, resulting in partial fertility, with 51.2%, 21.7%, and 23.4%, respectively (Fig. 6F, Supplementary Table S2 available at *JXB* online). The expression of *CsGAMYB1* in these eight transgenic plants was also analysed (Fig. 6G). We found that *CsGAMYB1* mRNA expressions in the five sterile lines were more than 3.4-fold higher than those in the three partial sterile plants. Line 8, which had only 1.9% fertility, displayed the highest level of *CsGAMYB1* mRNA. In line 8, *CsGAMYB1* mRNA level was more than 35-fold higher than that in line 1, in which the fertility reached 51.2%. This correlation between high levels of *CsGAMYB1* mRNA and sterility in multiple transgenic lines suggests that constitutive overexpression of *CsGAMYB1* results in male sterility in *Arabidopsis* in a dose-dependent manner.

### *CsGAMYB1* can regulate sex expression of cucumber via an ethylene-independent pathway

**Fig. 7.**
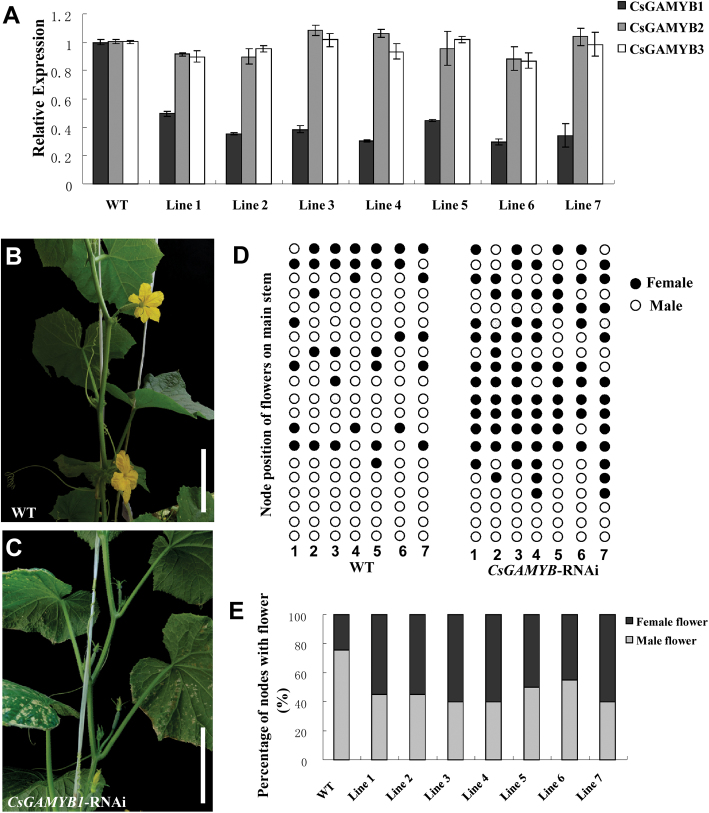
Sex expression of the flowers in the *CsGAMYB1*-RNAi plants of cucumber. (A) qRT-PCR analyses of *CsGAMYB1*, *CsGAMYB2*, and *CsGAMYB3* in wild-type (WT) plants and transgenic RNAi lines. The cucumber *α-TUBULIN* (*TUA*) was used as an internal control, and the experiments were repeated in three times. Error bars indicate the standard errors. (B, C) Phenotypes of WT (B) or transgenic RNAi plants (C) which are both 60 d old. (D) Diagrammatic data of sex expression of the flowers on the first 20 nodes of the main stems in WT and transgenic RNAi lines. The black and white circles represent female and male flowers, respectively. The numbers indicate the various transgenic lines. (E) The percentage of the nodes with male or female flowers up to the 20th node on the main stem of WT and transgenic RNAi lines. (This figure is available in colour at *JXB* online.)

To further determine the biological function of *CsGAMYB1* in cucumber, a double-strand RNAi construct containing the specific sequence of *CsGAMYB1* under the control of 35S promoter was introduced into the monoecious cucumber plants. A total of seven independent RNAi lines were obtained. In these RNAi lines, the transcript levels of *CsGAMYB1* were significantly reduced, whereas the expressions of the other two *GAMYB*-like genes, *CsGAMYB2* and *CsGAMYB3*, had no change as compared with those in the wild-type plants (Fig. 7A). This suggests that *CsGAMYB1* expression was effectively knocked down by RNAi, and this process had no effect on the expression of other *GAMYB*-like genes that show high sequence similarity with *CsGAMYB1*.

When *CsGAMYB1*-RNAi plants grew until anthesis of flowers on node 20, the sex of the flowers on each node of the main stem was recorded (Fig. 7B–E). The percentage of nodes with male flowers was 75.7% in wild type, and 45% in the *CsGAMYB1*-RNAi plants, whereas the proportion of nodes with female flowers was 24.3% and 55%, respectively. So, the ratio of nodes with male and female flowers in the transgenic RNAi plants decreased by almost 4-fold in comparison with that in wild-type, indicating that *CsGAMYB1* may promote male tendency or inhibit female tendency, leading to a significant change in the sex expression of flowers in cucumber.

**Fig. 8.**
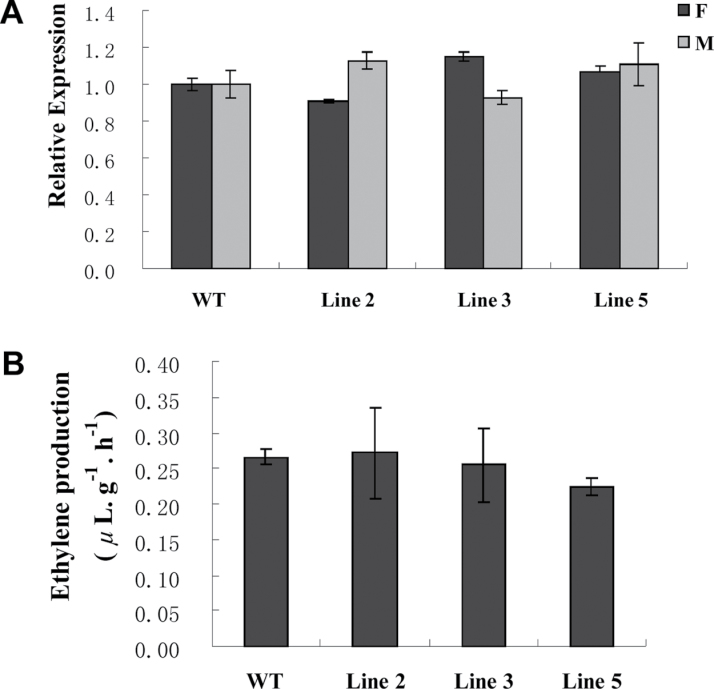
Ethylene is not involved in *CsGAMYB1*-regulated sex expression of cucumber. (A) Expression of *F* (*CsACS1G*) and *M* (*CsACS2*) by qRT-PCR in WT and transgenic RNAi lines. The cucumber *α-TUBULIN* (*TUA*) was used as an internal control, and the experiments were repeated in triplicate independent samples. Error bars indicate the standard errors. (B) Quantification of ethylene released from shoot apices in WT and transgenic RNAi lines at 4-leaf stage. Amount of ethylene was measured per 1g fresh weight and per h. Vertical bars indicate the standard errors of the mean for triplicate samples. (This figure is available in colour at *JXB* online.)

It has been reported that accumulation of *F* (*CsACS1G*) and *M* (*CsACS2*) mRNA and evolution of ethylene can control the sex determination of cucumber. To test whether there is a correlation between *CsGAMYB1*-regulated and ethylene-modulated sex expression of cucumber, we measured the expression of *F* and *M* genes and ethylene production in the shoot apices of *CsGAMYB1*-RNAi plants at the 4-leaf stage. As shown in Fig. 8, in transgenic lines 2, 3, and 5, neither *F* and *M* mRNA levels nor ethylene production had any significant changes as compared with wild type (Fig. 8A and B), suggesting that ethylene may not be involved in the pathway of *CsGAMYB1*-regulated sex expression of cucumber.

## Discussion

### *CsGAMYB1* may be the homologue for both *AtMYB33* and *AtMYB65*

In *Arabidopsis*, the *GAMYB* family has three members: *MYB33*, *MYB65*, and *MYB101* (Gocal *et al.*, 2001), in which *MYB33* and *MYB65* are functionally redundant in anther development (Millar and Gubler, 2005). Although in cucumber, there are also three putative *GAMYB*-like genes: *CsGAMYB1*, *CsGAMYB2*, and *CsGAMYB3*. *CsGAMYB1* is closely related to *AtMYB33* and *AtMYB65*, whereas *CsGAMYB2* and *CsGAMYB3* may not belong to the *GAMYB* family (Fig. 1B). Similar to AtMYB33 and AtMYB65, CsGAMYB1 also has an R2R3 repeat DNA-binding domain and three conserved motifs containing Box 1, Box 2, and Box 3 (Supplementary Fig. S1 available at *JXB* online). In addition, *CsGAMYB1* can partially rescue the phenotypes of *myb33 myb65* double mutant in *Arabidopsis*, with respect to stamen development and plant fertility (Fig. 5). These observations suggested that *CsGAMYB1* acts as the homologue for both *AtMYB33* and *AtMYB65* in cucumber.

### *CsGAMYB1* has both conserved and divergent functions with its hermaphroditic homologues

In hermaphroditic plants, *GAMYB* has been shown to play an important role in stamen and anther development. For example, *HvGAMYB* in barley, *OsGAMYB* in rice, and *AtMYB33* and *AtMYB65* in *Arabidopsis* were strongly expressed in floral organs, especially in stamens and anthers, but weakly in mature pollen grains, and the expression was upregulated by exogenous GA_3_ (Aya *et al.*, 2009; Gocal *et al.*, 2001; Kaneko *et al.*, 2004; Murray *et al.*, 2003; Tsuji *et al.*, 2006). Loss of function of *OsGAMYB* in rice, and *AtMYB33* and *AtMYB65* in *Arabidopsis* resulted in abnormal staminate specific organs, such as shorter filaments and pollen abortion caused by endless expansion of the tapetum, leading to male sterility (Alonso-Peral *et al.*, 2010; Aya *et al.*, 2009; Kaneko *et al.*, 2004; Liu *et al.*, 2010; Millar and Gubler, 2005), whereas overexpression of *HvGAMYB* in barley also led to decreased anther length and male sterility (Murray *et al.*, 2003). This suggests that *GAMYB* homologues have a conserved role in staminate development, but the regulatory mechanism may be different in various species. In our study, *CsGAMYB1* was highly expressed in male flower buds, where its expression was also up-regulated by GA_3_ application (Fig. 2), and *CsGAMYB1* rescued the phenotypes of *Arabidopsis* double mutant *myb33 myb65* in stamen development and plant fertility (Fig. 5, Supplementary Table S1 available at *JXB* online), indicating that *CsGAMYB1* may also function as a positive regulator for staminate development as those of *Arabidopsis MYB33* and *MYB65*. Meanwhile, ectopic expression of *CsGAMYB1* in *Arabidopsis* wild type resulted in reduced filaments, aborted pollen, and male sterility (Fig. 6, Supplementary Table S2 available at *JXB* online), similar to overexpression of *HvGAMYB* in barley, supporting the hypothesis that *CsGAMYB1* has a conserved role in staminate development.

However, unlike the weak expression of *HvGAMYB*, *OsGAMYB*, *AtMYB33*, and *AtMYB65* in mature pollen grains, *CsGAMYB1* is expressed throughout male flower development containing the mature pollen grains in cucumber (Fig. 3), but there are no significant difference in anthers and pollen development of male flowers between *CsGAMYB1*RNAi and wild-type plants in cucumber (data not shown), which is divergent with the phenotypes of the *GAMYB*deficient mutants in hermaphroditic species. Moreover, *GAMYB* genes in *Arabidopsis* might mediate GA signalling in flowering responses by activating the expression of a floral meristem identity gene, *LEAFY* (Gocal *et al.*, 2001). Also, in the grass *Lolium temulentum*, the content of GAs increases in the leaves and SAM (shoot apical meristem) with exposure to long-day which is sufficient to induce flowering (King *et al.*, 2001), and the increase in GAs level is followed by increased expression of *LtGAMYB* in the shoot apex, suggesting that *LtGAMYB* plays an important role in GA-regulated flower initiation (Gocal *et al.*, 1999). But in cucumber, even though high expression was detected in the inflorescence meristem (im) and floral meristem (fm) (Fig. 3A), flowering time of either male or female flowers seemed to be undisturbed upon partial loss of function of *CsGAMYB1* in the transgenic RNAi plants, indicating that *CsGAMYB1* may not be involved in floral initiation of cucumber. These observations verified that monoecious *CsGAMYB1* displays conserved as well as divergent functions with its hermaphroditic homologues.

### The possible roles of *CsGAMYB1* in *Arabidopsis*

Ectopic expression of *CsGAMYB1* in *Arabidopsis* leads to reduced filament length, aborted pollen, and male sterility (Fig. 6), and it is probably caused by a number of events. For example, *CsGAMYB1* may interfere with some *MYB* transcription factors of *Arabidopsis* that have been shown to be needed in GA-regulated stamen development (Cheng *et al.*, 2009; Millar and Gubler, 2005). Alternatively, *CsGAMYB1* possibly up-regulates the target genes of these *MYB* transcription factors and there may be feedback in this process. In addition, it could result from an accumulated level of GA signalling, which mimics the effect of GA overdose in flower development and male fertility (Colombo and Favret, 1996; Jacobsen and Olszewski, 1993). Interestingly, this developmental phenomenon owing to GA signalling is also observed in other plants. For instance, constitutive overexpression of *HvGAMYB* in barley causes male sterility (Murray *et al.*, 2003), whereas loss of function of *RGA* and *GAI*, repressors of GA signalling and plant growth and development, results in shorter stamen filaments and reduced pollen levels as well as fertility in *Arabidopsis* (Dill and Sun, 2001). Overall, despite ectopic expression of *CsGAMYB1* in *Arabidopsis* having an important role in stamen development, the regulatory mechanism is unclear, and further analysis is needed.

Moreover, *CsGAMYB1* can partially rescue the fertility of a *myb33 myb65* double mutant, and the recovery levels rely on the levels of *CsGAMYB1* expression (Fig. 5), whereas constitutive overexpression of *CsGAMYB1* results in male sterility in *Arabidopsis* (Fig. 6), suggesting that although *CsGAMYB1* is important for stamen development, the effect is dose dependent. And in the transgenic plants overexpressing *CsGAMYB1* in *Arabidopsis*, the expression of *CsGAMYB1* in line 8 is higher than that in line 4, 5, 6, and 7, but they are all male sterile (Fig. 6F and G, Supplementary Table S2 available at *JXB* online), indicating that the level of expression of *CsGAMYB1* in line 4, 5, 6, and 7 is sufficient to lead to sterility.

### GA and ethylene may regulate sex expression of cucumber via two parallel pathways

The sex determination of cucumber occurs owing to the selective arrestment of either carpel or stamen development at the bisexual stage (Bai *et al*., 2004; Malepszy and Niemirowicz-Szczytt, 1991). In the pathway of ethylene-regulated female sex expression, *F* gene governs female flowers formation (Knopf and Trebitsh, 2006; Mibus and Tatlioglu, 2004; Trebitsh *et al.*, 1997), whereas *M* gene inhibits stamen development in flower buds (Yamasaki *et al.*, 2001; Yamazaki *et al.*, 2003). *CsGAMYB1* can enhance the ratio of nodes with male and female flowers (Fig. 7), probably owing to either promotion in male tendency or repression of female sex expression. However, even though *CsGAMYB1* is highly expressed in both stamen primordia and carpel primordia at the bisexual stage, its expression remains high in the male specific organs, but weak in female flowers during later stages (Figs 2 and 3), suggesting that *CsGAMYB1* may promote the development of male flowers and inhibit the development of female flowers. In addition, in the *CsGAMYB1*-RNAi plants, the proportion of nodes with male flowers and female flowers is reduced and increased, respectively (Fig. 7), and no bisexual flowers are observed (data not shown). So, we conclude that *CsGAMYB1* may modulate the sex expression of cucumber by promoting male tendency and inhibiting the formation of female flowers at the same time, which potentially is an overlapping role with both *F* and *M* genes, even though the specific functions are opposite. Moreover, in the process of male flower development, *CsGAMYB1* expresses ubiquitously in the male flower, including sepal, petal, and stamen (Fig. 3), but has no effect on the male floral patterning when *CsGAMYB1* is knocked down by RNAi (data not shown), which possibly results from a number of events, one of which may be partial functional redundancy of three *CsGAMYBs* of cucumber. Although phylogenetic analysis has suggested that *CsGAMYB2* and *CsGAMYB3* may not belong to the *GAMYB* family (Fig. 1B), they show high similarity compared with *CsGAMYB1* (data not shown). Given that *Arabidopsis GAMYBs* have specific as well as partially overlapping roles, we speculate that *CsGAMYB1* may regulate male sex expression of cucumber specifically, whereas the three *CsGAMYBs* are likely to be functionally redundant in the development of male floral organs. However, for elucidating the functional similarities and differences among these three *CsGAMYBs*, the functional analysis of *CsGAMYB2* and *CsGAMYB3* in cucumber is the best way to elucidate this in future studies.

Sex differentiation of cucumber exists plasticity, and male and female expression can be changed by GA and ethylene, respectively (Iwahori *et al.*, 1969; MacMurray and Miller, 1968; Pike and Peterson, 1969; Wittwer and Bukovac, 1962); however, whether there is a crosstalk between these two pathways is unknown. In our study, we certified that GA can modulate sex expression of cucumber via the transcriptional regulation of *CsGAMYB1*, which acts a positive factor of GA signalling pathway (Figs 2B and 7), and this process has no effect on ethylene biosynthesis and production (Fig. 8), indicating that GA*-CsGAMYB1*-regulated male sex expression and ethylene-modulated female sex expression of cucumber might take two independent pathways. Our study on GA*-CsGAMYB1* reveals a new model for sex expression, which enhances our understanding of sex determination in cucumber and provides the basis for molecular flower induction and high-yield cultural practices. Besides, given that the signalling pathway GA–GID1–DELLA complex play important roles in plant growth and development, particularly in staminate development (Cheng *et al.*, 2004; Dill and Sun, 2001; Fleet and Sun, 2005; Griffiths *et al.*, 2006; Hou *et al.*, 2008; Sun, 2010; Sun, 2011; Tyler *et al.*, 2004), and *GAMYB* acts as an important downstream component of the DELLA proteins in *Arabidopsis* (Achard *et al.*, 2004; Fleet and Sun, 2005; Olszewski *et al.*, 2002), identifying the position of *CsGAMYB1* in GA response pathways and the relationship between *CsGAMYB1* and the GA–GID1–DELLA complex will shed light on the molecular mechanism of male sex expression of cucumber regulated by GA signalling.

### Accession numbers

Sequence data of GAMYB proteins in this study can be found in the Cucumber Genome DataBase, *Arabidopsis* Genome Initiative, Phytozome or GenBank/EMBL/Swiss-Prot databases under the following accession numbers: *CsGAMYB1* (Csa009014), *CsGAMYB2* (Csa019830), *CsGAMYB3* (Csa013555), *AtMYB33* (AT5G06100), *AtMYB65* (AT3G11440), *Malus domestica* (MDP0000147309), *Prunus persica* (ppa003628m.g), *Populus trichocarpa* (Potri.003G189700), *Manihot esculenta* (cassava4.1_004606m.g), *Gossypium raimondii* (Gorai.009G301100), *Glycine max* (Glyma13g25716), *Phaseolus vulgaris* (Phvul.011G191300), *Ricinus communis* (29686.t000037), *Solanum tuberosum GAMYB2* (PGSC0003DMG400005918), *Solanum tuberosum GAMYB1* (PGSC0003DMG400022689), *Solanum lycopersicum* (Solyc06g073640.2), *Oryza sativa* (Os01g59660), *Sorghum bicolor* (Sb03g037680), *Zea mays* (GRMZM2G139688), *Panicum virgatum* (Pavirv00069350m.g), *Hordeum vulgare* (AAG22863), *Lolium temulentu* (AAD31395).

## Supplementary data

Supplementary data are available at *JXB* online

Fig. S1. Sequence alignment of amino acid residues of CsGAMYB1 with other GAMYB proteins.

Table S1. *CsGAMYB1* can partially rescue the fertility of *myb33myb65* in *Arabidopsis*.

Table S2. Overexpression of *CsGAMYB1* resulted in partial sterility in *Arabidopsis*

Table S3. List of primers and their uses.

Supplementary Data
