# Supplementary material for: A GAMYB homologue CsGAMYB1 regulates sex expression of cucumber via an ethylene-independent pathway
Source: J Exp Bot. 2014 Apr 30;65(12):3201–13. doi: 10.1093/jxb/eru176 (PMC4071842; doi:10.1093/jxb/eru176)
Supplement: Supplementary Data [file supp_65_12_3201__index.html]

A GAMYB homologue CsGAMYB1 regulates sex expression of cucumber via an ethylene-independent pathway — A GAMYB homologue CsGAMYB1 regulates sex expression of cucumber via an ethylene-independent pathway — Supplementary Data 

# A *GAMYB* homologue *CsGAMYB1* regulates sex expression of cucumber via an ethylene-independent pathway

## Supplementary Data

Data files

**Files in this Data Supplement:**

- Supplementary Data - Supplementary Data
