# Supplementary material for: A GAMYB homologue CsGAMYB1 regulates sex expression of cucumber via an ethylene-independent pathway
Source: J Exp Bot. 2014 Apr 30;65(12):3201–13. doi: 10.1093/jxb/eru176 (PMC4071842; doi:10.1093/jxb/eru176)
Supplement: Supplementary Data [file supp_eru176_jexbot118372_file001.pdf]

Title: A *GAMYB* homolog *CsGAMYB1* regulates sex expression of cucumber via an ethylene-independent pathway

Yan Zhang[1,2], Xiaolan Zhang[1,2], Bin Liu[1,2], Wenjiao Wang[1,2], Xingwang Liu[1,2], Chunhua Chen[1,2], Xiaofeng Liu[1,2], Sen Yang[1,2], Huazhong Ren[1,2, ‡]

Supplementary material

Figure S1. Sequence alignment of amino acid residues of CsGAMYB1 with other GAMYB proteins.

Table S1. *CsGAMYB1* can partially rescue the fertility of *myb33myb65* in *Arabidopsis*.

Table S2. Overexpression of *CsGAMYB1* resulted in partial sterility in *Ar*abidopsis

Table S3. List of primers and their uses.

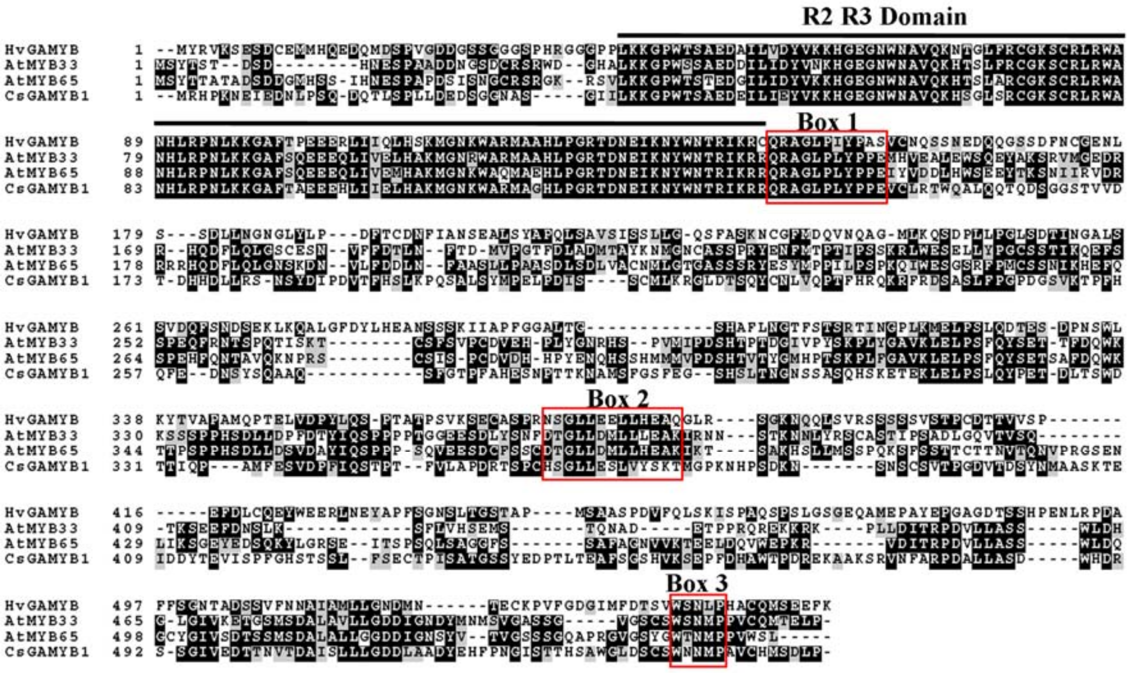

Figure S1. Sequence alignment of amino acid residues of CsGAMYB1 with other GAMYB proteins. The identical and similar residues are shown in black and gray, respectively. The highly conserved R2R3 domain and Boxes 1, 2, 3 are indicated in the black line and red boxes, respectively. Hv, *Hordeum vulgare*.

Table S1 *CsGAMYB1* can partially rescue the fertility of *myb33myb65* in *Arabidopsis*

Genotype		Total Number of Siliques/Plant	Number of Filled Siliques/Plant	Percentage of Filled Siliques (%)
myb33myb65		228.1±17.4	6.3±1.1	2.8±0.5
35S:CsGAMYB1 myb33myb65	Line 1	220	60	27.3
	Line 2	227	70	30.8
	Line 3	220	63	28.6
	Line 4	255	84	32.9
	Line 5	208	54	26.0
	Line 6	228	60	26.3
	Line 7	261	120	46.0
	Line 8	233	180	77.3

The values shown are the means ± SE of more than 10 plants from *myb33myb65*.

Table S2 Overexpression of *CsGAMYB1* resulted in partial sterility in *Ar*abidopsis

Genotype		Total Number of Siliques/Plant	Number of Filled Siliques/Plant	Percentage of Filled Siliques (%)
Col		114.8±9.9	105.9 ±9.8	92.2±1.7
35S:CsGAMYB1 Col	Line 1	127	65	51.2
	Line 2	106	23	21.7
	Line 3	111	26	23.4
	Line 4	118	5	4.2
	Line 5	105	5	4.8
	Line 6	97	2	2.1
	Line 7	114	3	2.6
	Line 8	103	2	1.9

The values shown are the means ± SE of more than 10 plants from *Col*.

Table S3 List of primers and their uses

Primer	Sequence	Used for
CsGAMYB1-F	5'-ATGCGGCATCCGAAAAATGAGA-3'	Cloning of *CsGAMYB1*
CsGAMYB1-R	5'-TCAAGGAAGATCAGACATATGACATACT-3'	
q-CsGAMYB1-F	5'-TCTAACCCTACCACAAAGAACGC-3'	qRT-PCR of *CsGAMYB1*
q-CsGAMYB1-R	5'-TCTATCTGGTGCCAACACAAAAGT-3'	
CsGAMYB1-SP6	5'-GATTTAGGTGACACTATAGAATGCTTAACCCTACCACAAAGAACGCC-3'	In situ probes of *CsGAMYB1*
CsGAMYB1-T7	5'-TGTAATACGACTCACTATAGGGGCTGAATGTGTTGTTGAAATCCC-3'	
G-CsGAMYB1-F	5'-GGAATTCATGCGGCATCCGAAAAATGAGA-3'	CsGAMYB1-GFP construct
G-CsGAMYB1-R	5'-CGGGATCCCGAGGAAGATCAGACATATGACATACT-3'	
O-CsGAMYB1-F	5'-GAAGATCTGATGCGGCATCCGAAAAATGAGA-3'	*CsGAMYB1* overexpression construct
O-CsGAMYB1-R	5'-GGACTAGTTCAAGGAAGATCAGACATATGACATACT-3'	
I-CsGAMYB1-F1	5'-AGGCGCGCCTACCGAGGTTATTTCTCCATTTG-3'	*CsGAMYB1*-RNAi construct
I-CsGAMYB1-R1	5'-CGATTTAAATTGACATACTGCTGGCATGTTATT-3'	
I- CsGAMYB1-F2	5'-GACTAGTTACCGAGGTTATTTCTCCATTTG-3'	
I-CsGAMYB1-R2	5'-CGGGATCCTGACATACTGCTGGCATGTTATT-3'	
CsACS1G-F	5'-AGAGGAGTAACGAAGACGAGGAAGT-3'	qRT-PCR of CsACS1G
CsACS1G-R	5'-CCACTCACCAACATTTTCTGTCTTT-3'	
CsACS2-F	5'-GAAAACTGTGAGGGAGAAGGGAA-3'	qRT-PCR of CsACS2
CsACS2-R	5'-GATAGTAAGGAGTGGGGACAAGCA-3'	
TUA-F	5'-ACGCTGTTGGTGGTGGTAC-3'	Internal controls in cucumber
TUA-R	5'-GAGAGGGGTAAACAGTGAATC-3'	
Actin2-F	5'-CCTTCGTCTTGATCTTGCGG-3'	Internal controls in *Arabidopsis*
Actin2-R	5'-AGCGATGGCTGGAACAGAAC-3'	
